# Nonlinear analysis of biomagnetic signals recorded from uterine myomas

**DOI:** 10.1186/1477-044X-4-2

**Published:** 2006-03-30

**Authors:** Athanasia Kotini, Achilleas N Anastasiadis, Photios Anninos, Nikoleta Koutlaki, Panagiotis Anastasiadis

**Affiliations:** 1 Lab of Medical Physics and Department of Obstetrics and Gynaecology, Medical School, Democritus University of Thrace, University Campus, Alexandroupolis, Greece

## Abstract

**Objective:**

To determine if there is any non-linearity in the biomagnetic recordings of uterine myomas and to find any differences that may be present in the mechanisms underlying their signal dynamics.

**Methods:**

Twenty-four women were included in the study. Sixteen of them were characterised with large myomas and 8 with small ones. Uterine artery waveform measurements were evaluated by use of Pulsatility Index (PI) (normal value PI<1.45).

**Results:**

Applying nonlinear analysis to the biomagnetic signals of the uterine myomas, we observed a clear saturation value for the group of large ones (mean = 11.35 ± 1.49) and no saturation for the small ones.

**Conclusion:**

The comparison of the saturation values in the biomagnetic recordings of large and small myomas may be a valuable tool in the evaluation of functional changes in their dynamic behavior.

## Introduction

Several aspects of nonlinear dynamics have been explored in physics and medical engineering in recent years [1]. Descriptions of linear and nonlinear systems, fractal geometry, and neural networks are all relevant to the study of oncological problems. The complexity of biological processes is associated with interrelationships between many different factors, and mathematical simplifications via mechanical models can help elucidate biological behavioral patterns, whether on genetic, cell, tissue, tumor, organ, or whole-body scales. Dynamics describes systems that change in space and time – this is also the nature of biological processes [2,3]. Cancer reflects dynamical and multistage processes. Oncogenes gain a malignant function through mutation or chromosomal defect or through viral acquisition into the genome. Tumor suppressor genes promote neoplasia as a consequence of the loss of their normal regulation. Tumors may exhibit an increase in complexity (e.g., tumor promoter genes) or a decrease in complexity (e.g., allelic loss and tumor suppression genes) [4].

No previous studies have applied nonlinear dynamic analysis to biomagnetic activity of uterine myomas. Therefore, in this pilot study we measured the complexity of uterine myomas activity in to ascertain whether nonlinear dynamics would allow discrimination of large from small ones.

## Methods

The study group consisted of 24 premenopausal women who were planed to undergo laparotomy because of symptomatic myomas. Sixteen of them were characterised with large myomas and 8 with small ones. The diagnosis of myomas was made by use of bimanual gynecologic examination as well as with both transabdominal and transvaginal gray-scale sonography. The transabdominal examination was necessary to adequately measure uterine size in women with large uterus. Transvaginal examination was performed with a woman in the lithotomy position. Myoma volume was expressed in cm^3 ^and was calculated according to the formula length (cm) × depth (cm) × width (cm) × 0.5. A myoma was considered large if at least one of its diameters was > 5 cm; otherwise it was characterized a small one. If more than one myoma was found in the pelvis, the largest myoma was examined. The uterine arteries and myoma vascularization were visualized by the color Doppler technique. Blood flow velocity waveforms from both uterine arteries were obtained by placing the Doppler gate over the colour areas and activating the pulsed Doppler function. The main stem of the uterine arteries was examined lateral to cervix at the level of the internal os. The mean value from the PI obtained from the right and left uterine artery of each patient was recorded and correlated with the myoma volume and with the corresponding biomagnetic measurements. All women underwent hysterectomy or excision of the myoma and histologic diagnosis of a benign uterine myoma was made for all of them.

Biomagnetic recordings were obtained by a single channel second order gradiometer DC-SQUID (MODEL 601; Biomagnetic Technologies Inc., San Diego, USA) [5-7]. During the recording procedure the patient was relaxed lying on a wooden bed free of any metallic object so as to decrease the environmental noise and get better signal to noise ratio. The recordings were performed after positioning the SQUID sensor 3 mm above the exact position of myomas in order to allow the maximum magnetic flux to pass through the coil with little deviation from the vertical direction. Five points were selected for examination according to the myoma topography made by use of gynecologic and ultrasound examination. Point 5 was located at the very center of the myoma, whereas points 1–4 were located at the periphery of the examined area. The measured magnetic field was at the order of 10^-12 ^Tesla. By convention the maximum of the 5 values was used when assessing each myoma. For each point 32 recordings of 1-second duration each were taken and digitized by a 12 bit precision analogue – to – digital converter with a sampling frequency of 256 Hz. The duration of the above recordings is justified because the chosen time interval is enough to cancel out, on the average, all random events and to allow only persistent ones to remain. The biomagnetic signals were band-pass filtered, with cut-off-frequencies of 0.1–100 Hz. The associated Nyquist frequency limit, with the above-mentioned sampling frequency, is therefore 128 Hz, which is well above the constituent frequency components of interest in biomagnetic recordings and avoids aliasing artifacts. Informed consent for the study was obtained from all the patients prior to the procedure.

### Theory and algorithm

Nonlinear analysis was used to estimate the complexity index of the strange attractor characterizing the biomagnetic time series obtained from the patient. According to Grassberger and Procaccia [8], the dynamics of the system can be experimentally reconstructed from the observed biomagnetic time series B_i _= B(t_i_) (i = 1, 2, ..., 8192) and the vector construction of V_i _is given by the following equation:

V_i _= {B_i_,B_i+τ_,...,B_i+(m-1)τ_}     (1)

This equation gives a smooth embedding of the dynamics in an m-dimensional phase space. The evolution of the system in the phase space – once transients die out – settles on a submanifold, which is a fractal set called the strange attractor. The strange attractor can be described by a geometrical parameter: the complexity index D. This parameter is related to the number of variables required to define the attractor within the phase space and it can be estimated from an experimental time series by means of the correlation integrals C(r,m) defined as follows:



where Θ (u) is the Heaviside function defined as Θ(u) = 1 for u>0 and Θ(u) = 0 for u≤ 0); m is the embedding dimension; n is the number of vectors constructed from a time series with N samples, and is given by the formula n = N-(m-1)τ (where τ is a delay parameter which is equal to the first zero crossing of the autocorrelation time of the biomagnetic signal); and B is a correction factor for spurious influences of autocorrelation [9]. The correlation integral C(r,m) measures the spatial correlation of the points on the attractor and it is calculated for different values of r in the range from 0 to r_max_, where r_max _is the maximum possible distance of two random selected points of the attractor of the selected time series. The r_max _is equal to (m)^1/2 ^(x_max _-x_min_), (assuming that x_max _and x_min _are the maximum and minimum recorded values in the time series). The complexity index D of the attracting submanifold in the reconstruction phase space is given by:



In the case of a chaotic signal exhibiting a strange attractor, there is a saturation value (plateau) in the graph of the function D vs. ln(r(2-r)). This value remains constant, although the signal is embedded in successively higher-dimensioned phase spaces. Using the above method the complexity index D of the selected time series was estimated for the biomagnetic activity of the large and small myomas.

## Results

**Figure 1:**
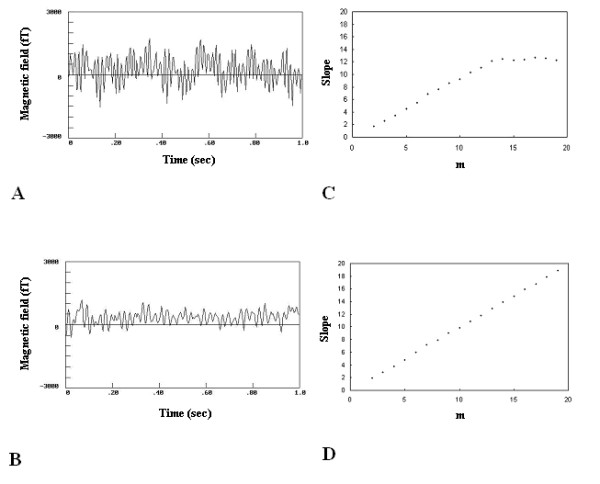
A) The wave-form of the magnetic field emitted from a woman with large myoma. B) The wave-form of the magnetic field emitted from a woman with small myoma. C) Slopes versus embedding dimensions reveal that there is a saturation value around 12 in the large myoma and D) absence of saturation in the small one.

Figure 1A shows the raw data of a large uterine myoma whereas Fig. 1B of a small one. The raw data were of high amplitudes in the large uterine myomas and of low amplitudes in the small ones. Figure 1C shows the estimated complexity indices of the slopes versus embedding dimensions in a large myoma and Fig 1D in small one. A clear saturation value around 12 was observed for the large myoma and no saturation for the small one.

Table 1 presents the mean values of the myoma volume, the uterine artery PI and the saturation values in the two study groups. A statistically significant difference was observed in the PI values obtained from large and small myomas respectively (P < 0.0005). Higher PI values were recorded in the uterine arteries of uteri with small myomas, while lower PI values were observed in the uterine arteries supplying large myomas. There was a saturation value (mean = 11.35 ± 1.49) in the group of large myomas and absence of saturation in the group of small ones.

**Table 1:** Mean values of myoma volume, uterine artery PI and saturation values in the two study populations.

	**Volume**	**PI**	**Saturation values**
**Large myomas (16 cases)**	287.19 (99.8–1119.4)	43.11 (29.6–56.25)	11.35 ± 1.49
**Small myomas (8 cases)**	1.06 (0.67–1.29)	2.02 (1.19–2.76)	ABSENCE
**P**	<0.0005	<0.0005	-

## Discussion

According to nonlinear dynamical system [10], the dynamics of any physical or biological system can be quantified and described in terms of the motion of an attractor (e.g., strange or chaotic) and complexity indices of the reconstructed phase space. These concepts reflect some geometrical properties of the reconstructed phase space of the dynamical system under consideration, and these can be extracted. It is of great importance in the non linear analysis of a dynamical system the existence of low-dimension attractors and the estimation of the complexity indices D of the attractors [11-15].

The difficulty of applying fractal mathematical processes to tumor biology is clear from the paucity of models that are useful to clinical practice. Tumors are nevertheless unstable systems, as illustrated by their heterogeneity in tumor genetics, aneuploidy, morphology, and growth patterns, for example. The change from cumulative minor genetic dysfunctions to catastrophic malignancy may thus have mathematical parallels. If tumors do exhibit chaotic properties, we may find them in the structural morphology of the whole tumor or its component parts, in the dynamic growth characteristics, in their patterns of behavior, or even in their response to therapy. It is the instability associated with a tumor (e.g., volume increase, invasion, or metastasis) rather than the existence of the tumor which kills. The restoration of stability or steady-state symbiosis of a tumor with its neighboring tissues may be an important objective in therapeutics.

By comparing the complexity indices it can be observed that there is a clear saturation value in large myomas and an absence of saturation value in small ones. Such a difference reflects an increase in the parameters (less organization, more chaoticity), which are needed in order to describe their dynamics. In terms of pathophysiology of myomas the observed difference which appeared in the biomagnetic recordings of the small myomas can be expressed as a distortion of the high rhythmicity and synchronization which characterized the large versus small myomas. It seems therefore that biomagnetic measurements with the use of non-linear analysis may prove to be a useful method in differentiating large and small myomas. It is true that biomagnetometry needs special equipment, a suitable prepared room and a good methodological knowledge for the employed methods, but once we have these requirements the method is rewarding. It is a non-invasive procedure, reliable, rapid and easy to interpret. Furthermore, it is totally harmless and well tolerated by women.

Our data suggest that biomagnetic measurements and nonlinear analyses are optimal procedures for assessing and differentiating uterine myomas.
